# 

**Published:** 1893-05-13

**Authors:** 


No. 346, Vol. XIV.—May 13th, 1893.

[Registered at the General Post Office as a Newspaper for transmission in the United Kingdom and Abroad.]

WITH EXTRA NURSING SUPPLEMENT

Per Annum, 8s. 6d., post free.

Monthly Parts, 6d.; post free, 8d.

Ditto per Annum; 8s., post free.

